# Mapping global cancer risk hotspots from environmental pesticides

**DOI:** 10.1016/j.isci.2026.116599

**Published:** 2026-07-07

**Authors:** Yabi Huang, Zijian Li

**Affiliations:** 1 School of Biosafety, Shenzhen Campus of Sun Yat-sen University, Sun Yat-sen University, Shenzhen, Guangdong 518107, China

**Keywords:** pesticide risk management, human health, cancer risk assessment

## Abstract

Pesticides remain widely detected in the environment and continue to pose long-term cancer risks to human populations. Here, we developed a global framework to quantify population-level cancer risk (CR) from 22 carcinogenic pesticides by integrating contamination in air, soil, surface water, and groundwater with oral, inhalation, and dermal exposure pathways. Using global monitoring data from 2010 to 2020, we mapped CR values across 205 countries and regions under basic and advanced exposure scenarios. Risks show marked spatial heterogeneity, with hotspots in parts of Asia, Africa, and the Americas exceeding (CR > 10^−4^). Historical use, delayed bans, and ongoing releases explain these patterns. Lifetime average daily doses (LADDs) analysis indicates that water-related pathways contributed substantially under the advanced scenario, highlighting water pathways in cumulative cancer risk. Our results demonstrate that legacy pesticides still pose measurable cancer risks after being banned, underscoring the need for integrated, multi-pathway risk management strategies.

## Highlights

• A global framework quantified cancer risk from environmental pesticides
• Cancer risk hotspots exceed CR > 10^−4^ in parts of Asia, Africa, and the Americas
• Water-related exposure contributes substantially to lifetime average daily dose
• Results support integrated, multi-pathway approaches to pesticide risk management

## Graphical abstract

**Figure:**
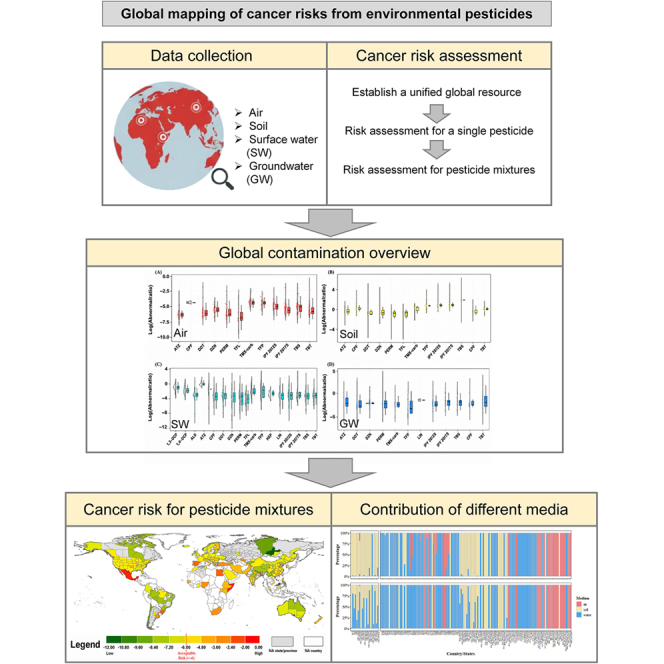

## Teaser

Immunology; Environmental science; Biological sciences; Genetics; Genomics; Neuroscience; Cancer

## Introduction

Pesticides are widely used chemicals deliberately introduced into the environment to improve agricultural productivity.^1^^,^^2^ However, their extensive application has led to pervasive contamination that extends far beyond treated fields. After application, pesticides may accumulate within food webs, volatilize into the atmosphere, leach into groundwater, or run off into surface waters and eventually the oceans, facilitating their movement across local, regional, and even transboundary scales.^3^^,^^4^^,^^5^^,^^6^ Consequently, pesticides have been detected worldwide, with monitoring data from numerous countries confirming their presence in various off-field environmental compartments.^7^^,^^8^

Some pesticides are carcinogenic and pose long-term health risks to humans.^9^^,^^10^^,^^11^ This issue is particularly critical for historically used pesticides that persist in the environment for decades after being banned. Owing to their chemical stability and bioaccumulative nature, these legacy pesticides can remain in soil, water, air, and biota, continuously contributing to human exposure. People may be exposed through multiple pathways, including ingestion of contaminated food, soil, or water; inhalation of polluted air or dust; and dermal contact with residues in soil or household environments.^9^^,^^12^^,^^13^^,^^14^ Accumulating evidence indicates that exposure to carcinogenic pesticides is linked to increased risks of various cancers in human populations.^15^ Therefore, assessing and managing the cancer risks associated with environmental pesticides is essential for safeguarding public health.

However, current approaches to assessing the health risks of carcinogenic chemicals have several limitations. Most existing frameworks evaluate risks based on a single exposure route, typically oral or inhalation, using toxicological parameters such as cancer slope factors (CSFs) to estimate lifetime cancer risks.^16^ While oral CSFs are relatively well-documented through animal bioassays and human extrapolations, inhalation unit risks and dermal CSFs remain unavailable for most pesticides.^17^ After entering via any route (such as oral, inhalation, or dermal), active pesticide ingredients undergo systemic transport, potentially accumulating across multiple organs and tissues. This systemic accumulation increases the likelihood of multi-organ interactions and cumulative tumor development, especially in sensitive or high-retention organs such as the liver, kidneys, and adipose tissue.^18^^,^^19^^,^^20^ Conventional risk assessment frameworks, which often treat each exposure route and target organ independently, may therefore underestimate the compound effects of chronic, multi-route pesticide exposure on carcinogenesis.^21^ This route-specific focus makes it difficult to capture the complexity of real-world exposure scenarios, in which individuals are simultaneously exposed through multiple pathways and routes.^22^^,^^23^ Although some studies have introduced relative potency factors to assess mixtures of carcinogenic chemicals with a common mode of action, substantial gaps remain in evaluating cumulative risks from mixtures that act through distinct mechanisms or target multiple organs.^24^ Consequently, current risk assessment methods may underestimate or mischaracterize the overall carcinogenic burden posed by environmental chemical mixtures, underscoring the need for more integrated, mechanistic, and cumulative approaches.^25^

Therefore, the aim of this study is to establish a methodological framework for assessing population-level cancer risks from environmental pesticides by considering all possible exposure pathways and pesticide species. The proposed framework is then applied in combination with global pesticide pollution data to map the worldwide distribution of pesticide-related cancer risks, providing a comprehensive overview of the global cancer risk burden to support future research and policy development.

## Results

### Global contamination overview of carcinogenic pesticides

To elucidate the global distribution of pesticides in the environment during the period 2010–2020, a total of 20,160 publications were retrieved and screened. Ultimately, data from 698 environmental studies were utilized to construct a comprehensive global dataset. Based on this dataset, this study focused on carcinogenic pesticides targeting individual organs and those with established toxicity parameters.^26^ They are 1,1,1,2-tetrachloroethane, lindane, aldrin, α-HCH (hexachlorocyclohexane), β-HCH, biphenyl, chlordane, DDD (dichlorodiphenyldichloroethane), dieldrin, heptachlor, heptachlor epoxide, hexachlorobenzene, kepone, *p*,*p'*-DDE (dichlorodiphenyldichloroethylene), *p*,*p'*-DDT (dichlorodiphenyltrichloroethane), prochloraz, toxaphene, 1,3-dichloropropene, aniline, hexachlorobutadiene, hexachloroethane, 1,4-dioxane. These selected pesticides include representative organochlorines and other legacy compounds that have been widely detected across environmental media despite regulatory bans or restrictions.^27^^,^^28^

**Figure 1:**
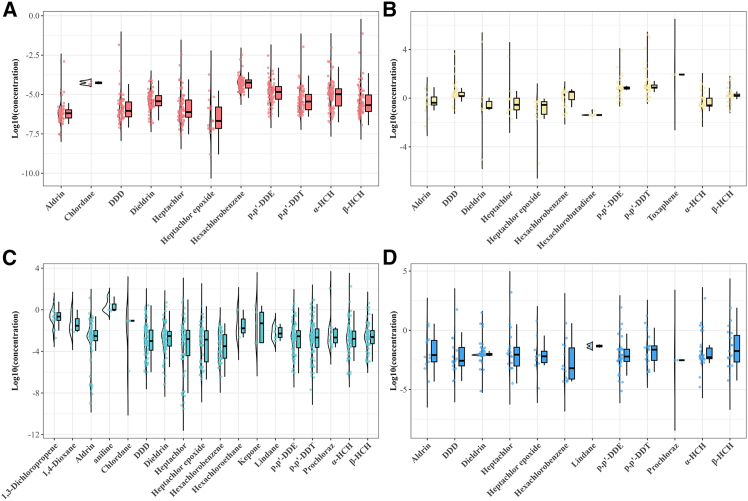
Concentration distributions of the selected pesticide in different environmental compartments (A) Air. (B) Soil. (C) Surface freshwater. (D) Groundwater. Boxes indicate the interquartile range (IQR), horizontal lines within the boxes indicate medians, and whiskers represent 1.5 × IQR. Points represent individual observations.

The data for the selected carcinogenic pesticides revealed widespread pesticide contamination across multiple environmental compartments, including air, soil, surface freshwater, and groundwater (Table S1). Figure 1 summarizes the distribution of log_10_-transformed concentrations for representative pesticides across countries and states. Across all four environmental compartments (air, soil, surface freshwater, and groundwater), the concentrations of the selected carcinogenic pesticides spanned 5–11 orders of magnitude, indicating substantial spatial heterogeneity at the global scale.

In the atmospheric compartment (Figure 1A), the concentrations for the selected pesticide were between 2.10 × 10^−13^ mg kg^−1^and 7.50 × 10^−8^ mg L^−1^, with the median values of 4.43 × 10^−12^ mg L^−1^. Nevertheless, detectable atmospheric levels were observed for ten selected pesticides, including aldrin, chlordane, dieldrin, heptachlor, heptachlor epoxide, hexachlorobenzene, hexachlorobutadiene, *p*,*p′-*DDE, *p*,*p′*-DDT, and HCH isomers. Several pesticides, such as aldrin and hexachlorobenzene, showed relatively narrow overall concentration ranges, while others exhibited much wider ranges with extremely high values. Pesticide concentrations in soil exhibited greater variability compared with those in air (Figure 1B). The concentrations of some pesticides, such as heptachlor, heptachlor epoxide, and *p*,*p'*-DDT, spanned five orders of magnitude. While hexachlorobutadiene displayed relatively constrained concentration ranges due to limited data, dieldrin spanned nine orders of magnitude, with the values from 9.00 × 10^−9^ mg kg^−1^ to 42.5 mg kg^−1^.

In aquatic environments, both surface freshwater and groundwater showed widespread contamination (Figures 1C and 1D). Pesticide concentrations in surface freshwater generally exhibited broader distributions than in the air and soil, with median values typically between 1.52 × 10^−6^ mg L^−1^ and 8.80 × 10^−5^ mg L^−1^, and upper extremes reaching 1 × 10^−4^ mg L^−1^ for some pesticides. Groundwater concentrations were similar, with medians commonly between 2.70 × 10^−6^ mg L^−1^and 4.90 × 10^−5^ mg L^−1^, and displayed substantial variability across regions.

Overall, the observed concentration patterns demonstrate that the selected 22 carcinogenic pesticides are globally ubiquitous, multimedia in distribution, and characterized by pronounced regional heterogeneity. These quantitative contamination profiles provide a critical empirical basis for subsequent multimedia exposure integration and population-level cancer risk assessment.

### Cancer risk assessment for environmental pesticides

Based on the concentration data, this study conducted a carcinogenic health risk assessment for the cumulative human exposure to pesticide residues from four environmental media using an integrated approach (Table S1). According to US EPA guidelines, CR values below 1 × 10^−6^ (log_10_CR < −6.00) mean the cancer risks are negligible, values between 1 × 10^−6^ and 1 × 10^−4^ (−6.00 ≤ log_10_CR ≤ −4.00) are deemed acceptable risk, and values exceeding 1 × 10^−4^ (log_10_CR > −4.00) indicate unacceptable risk. In this study, regions with CR or TCR values exceeding 1 × 10^−4^ were defined as hotspots.

**Figure 2:**
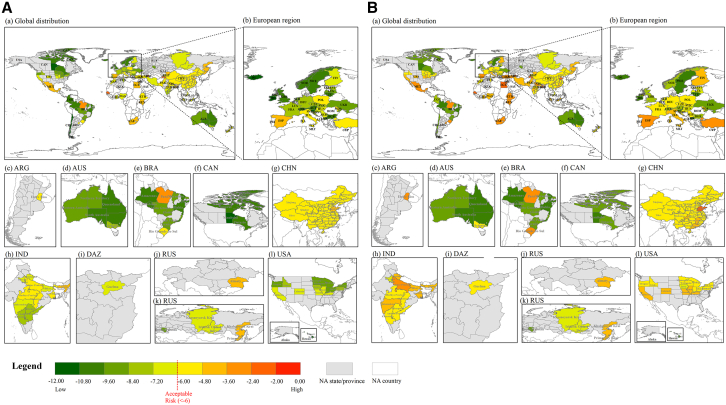
Log-transformed cancer risk maps for p,p'-DDT exposure (A) Basic scenario. (B) Advanced scenario.

In this subsection, p,p*′*-DDT was selected as a representative carcinogenic pesticide for further analysis due to its environmental persistence and data availability. The assessment was performed under two distinct exposure scenarios: a basic scenario (conservative assumptions) and an advanced scenario (incorporating refined environmental fate and bioaccessibility parameters). The resulting geographic distributions of log-transformed CR are presented in Figures 2A and 2B, respectively. In addition, the CR values summary statistics and high-risk hotspots are listed in Table S2. Under the basic exposure scenario, the estimated log-transformed CR associated with human exposure to *p*,*p′*-DDT exhibited pronounced spatial heterogeneity at both global and subnational scales. At the global level, log_10_(CR) spanned several orders of magnitude, with values ranging from below −12.64 in low-contamination regions to values approaching −1.36 in certain countries. The areas with log_10_(CR) higher than −6 were predominantly clustered in Asia and Africa, with the median log_10_(CR) value of −6.18 and −5.70, respectively. At the national scale, China (Figures 2A–2G) and India (Figures 2A–2H) showed the widespread high-risk zones, with the median log_10_(CR) value of −5.69 and −6.18, respectively. In contrast, countries including Australia, Canada, and the United States (USA) displayed predominantly low to moderate risk levels (Figures 2A–2D, 2F, and 2L). Under the advanced exposure scenario (Figure 2B), the proportion of land area where log_10_(CR) > −6.00 was substantially increased. For instance, the area with unacceptable risk in India increased substantially. The number of Indian states/union territories with log_10_(CR) exceeding −6 increased dramatically from 4 under the basic scenario to 11 under the advanced scenario (Figures 2A–2H vs. 2B–2H). The comparison between the two scenarios highlights the critical influence of considering comprehensive exposure patterns on the final characterization of health risks.

**Figure 3:**
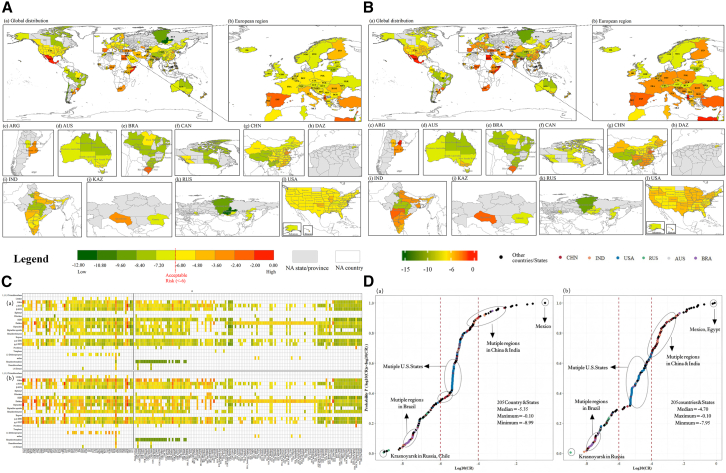
The log-transformed total cancer risk (TCR) values for population exposure to pesticide mixtures (A) The geographic distribution map of log-transformed TCR values under the basic scenario. (B) The geographic distribution map of log-transformed TCR values under the advanced scenario. (C) Log-transformed TCR values of the selected pesticides across multiple countries/states (a, basic scenario; b, advanced scenario). (D) The cumulative probability analysis for TCR values (a: basic scenario; b: advanced scenario).

In addition, the total cancer risk (TCR) values associated with population exposure to pesticide mixtures were assessed under the same scenarios (Figures 3A and 3B for basic and advanced scenarios, respectively), and the CR values for individual pesticides are shown in Figure 3C. Similarly, the TCR values summary statistics and high-risk hotspots are listed in Table S2. Under the basic scenario (Figure 3A), high-risk regions with log_10_(TCR) exceeding −6 were predominantly concentrated in parts of North America, Asia, and Africa. In contrast, most areas of Oceania displayed lower risk levels. The advanced scenario (Figure 3B) generally showed a similar global risk pattern, but with an overall increase in TCR levels, suggesting that the parameters and assumptions of the advanced scenario contributed to an escalated risk outcome. For example, in the USA, under the baseline scenario (Figures 3A–3L), risk values across most states were similar, ranging from 1.00 × 10^−10^ to 5.10 × 10^−8^. However, under the advanced scenario (Figures 3B–3L), risk levels increased significantly, with most states exceeding the acceptable risk threshold, and the median risk value reaching 1.40 × 10^−6^. In contrast, multiple regions in India (Figures 3A–3I) and China (Figures 3A–3G) consistently exhibited high TCR values in both assessments, indicating that the risk is widespread. The cumulative probability analysis (Figure 3D) demonstrated that the TCR values for aggregated regions like China and India were generally higher than the median observed across the 201 countries/states (e.g., median of −0.5278 in Figure 3D–a, −4.70 in Figure 3D–b), indicating a greater cumulative risk burden for the populations in these areas.

We conducted uncertainty analysis of model parameters, monitoring data gap, and aggregation assumptions for the TCR under the advanced scenario (Tables S3, S4, and S5). For parameter uncertainty analysis, although the absolute TCR values varied under lower-bound and upper-bound assumptions, the main hotspot regions remained broadly stable. Sensitivity analysis identified body weight, swimming intake rate, fish and shellfish consumption, and the bioconcentration factor (BCF) as the strongest influencers of the advanced-scenario TCR, while other parameters had smaller effects. Overall, these results suggest that the main cumulative-risk conclusions are robust. The sensitivity analysis based on alternative data-coverage thresholds showed that the principal hotspot pattern (especially in parts of Asia, Africa, and the Americas) remained broadly stable, although spatial coverage decreased as stricter inclusion criteria were applied. The median TCR increased from 2.0 × 10^−5^ in the baseline analysis to 7.2 × 10^−5^ in the moderate-coverage subset and 1.8 × 10^−4^ in the high-coverage subset. Excluding pooled multi-province/state records also reduced spatial coverage and lowered the median TCR from 2.0 × 10^−5^ to 1.8 × 10^−5^, but the hotspot pattern remained stable. Overall, these results suggest that data gaps and aggregation assumptions affect spatial coverage and the magnitude of risk estimates, but do not materially alter the main global high-risk pattern.

## Discussion

### Contribution of exposure pathways to total cancer risk

**Figure 4:**
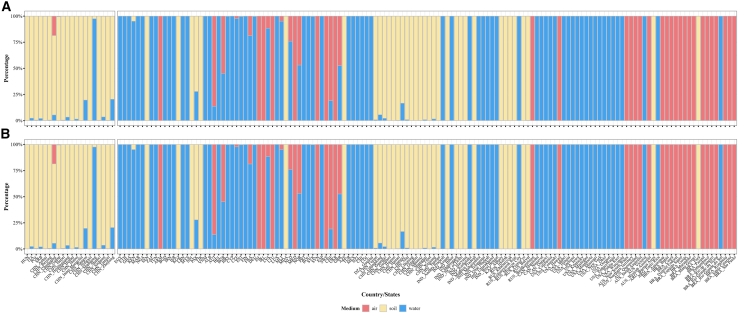
Relative contributions of different environmental compartments to the total cancer risk (TCR) across multiple countries/states Only the first 20 countries/states with detectable p,p′-DDT concentrations in all three environmental media are shown. (A) Basic scenario. (B) Advanced scenario.

The lifetime average daily dose (LADD) was calculated to link environmental pesticide concentrations with human carcinogenic risk. Figure 4 presents the LADD ratio for all three major environmental media across multiple countries/states. This section focuses on the subset of countries and regions for which LADD estimates were available simultaneously for all three major environmental media—air, soil, and water. Specifically, the discussion centers on the first 20 countries/states in Figure 4.

Under the basic exposure scenario, soil-related pathways of most countries/states contributed a substantial fraction of the total LADD. This pattern primarily reflects the relatively high pesticide concentrations retained in soils. Soils act as long-term sinks for many carcinogenic legacy pesticides with strong sorption capacity and long residence times, and accumulate residues from historical applications even decades after use cessation.^29^^,^^30^^,^^31^ For example, despite regulatory bans, organochlorine pesticides, such as DDT and its metabolites (DDE and DDD), lindane and other HCH isomers, were frequently detected at elevated concentrations in soils across multiple regions.^32^^,^^33^^,^^34^ Therefore, active management or remediation of contaminated soils is an effective strategy to yield a proportional reduction in cancer risk.^35^

In contrast, under the advanced exposure scenario, the contribution structure of LADD shifted markedly. Water-related exposure pathways in some countries emerged as a major contributor to total internal dose. This indicated that indirect exposure pathways such as dermal absorption and inhalation during showering, bathing, and recreational activities, which are commonly ignored in routine risk assessments, can play a critical role in shaping cumulative cancer risk. The enhanced contribution of water-related exposure pathways can be mechanistically interpreted using the physicochemical parameters summarized in Table S6. For instance, 1,3-dichloropropene, hexachlorobutadiene, and hexachloroethane have high air-water partition coefficients (K_aw,_ unitless) values of 1.05, 0.421, and 0.288, respectively. This suggests the potential for partial pesticide volatilization during domestic activities such as showering and bathing. In addition, the selected pesticides in this study have relatively high dermal uptake rates (Kp) with a range from 3.30 × 10^−4^ to 6.28 × 10^−1^ cm h^−1^, which enhanced the skin permeability of pesticides associated with domestic water use. Crucially, the importance of water-related pathways is further supported by the BCF (unitless), which ranged from 4.06 × 10^−1^ to 8.24 × 10^3^.

This process creates a secondary inhalation pathway that operates concurrently with dermal exposure, thereby increasing overall internal dose. High BCF values indicate an enhanced capacity of certain pesticides to transfer from the water into biological tissues, thereby increasing their bioavailability within aquatic systems. This property increases the potential for indirect human exposure associated with water bodies, including but not limited to dietary pathways.^36^^,^^37^^,^^38^ These findings demonstrate that water bodies should be treated as dynamic exposure interfaces linking aquatic contamination to oral, dermal, and inhalation uptake, which have important implications for cumulative cancer risk assessment.

Overall, this section reveals that some indirect exposure pathways traditionally considered secondary can substantially influence TCR.

### Spatial patterns of pesticide-related cancer risk and the role of legacy contamination

As described in previous sections, cancer risks had geographic hotspots. Most countries in Africa and Asia show markedly higher CR values than other continents under two assumed scenarios, including parts of North Africa and the Middle East, and South and Southeast Asia. These hotspots are likely explained by the high environmental residual levels, especially in settings where pesticides have been intensively used over long periods and where environmental conditions favor persistence and redistribution. In south Asia (e.g., Bangladesh and parts of India) and regions with dense agricultural activity, historical and ongoing pesticide inputs increase the likelihood of multi-media contamination.^39^ In addition to countries in south and southeast Asia, Mexico is also a regional hotspot with high CR values. Evidence indicates that DDT was widely used in Mexico for malaria control well into the 1990s, with the formal phase-out occurring around 2000s.^40^ This delayed ban implies a greater historical burden in Mexico compared with the European region or the USA, which banned around the 1970s.^41^ These persistent organochlorine pesticides can remain in soils and water for decades^42^^,^^43^ and subsequently re-enter air and water through volatilization, erosion, and hydrological transport, producing sustained exposure even when current use patterns change.^44^^,^^45^^,^^46^ Importantly, these patterns cannot be explained solely by contemporary pesticide use, but instead point to the enduring influence of legacy contamination. Notably, notwithstanding these spatial patterns, the CR results in this study should be interpreted with caution due to the dependence on data availability and representativeness. This limitation is particularly relevant for some African countries, such as Algeria, Senegal, and Somalia, where pesticide concentration data are limited.

To further explore the relationship between environmental contamination and cancer risk, we examined the correlation between a previously developed air-based^47^ and water-based^7^ pesticide pollution index (PI) and the country-level averaged cancer risk derived in this study. The calculation processes and scatterplots are shown in the (Figure S1 and Table S7), respectively. No significant correlation was observed between PI in air and CR under either the basic or advanced exposure scenarios (Spearman’s rank correlation, r = −0.0477 and −0.121, respectively; *p* > 0.1). This lack of association indicates that pesticide-related cancer risk cannot be explained by air contamination alone. It is reasonable because cumulative cancer risk emerges from the integration of multiple environmental compartments and exposure pathways rather than from contamination in the air. Beyond the cumulative nature of the risk metric, this weak association likely reflects the fact that general-population pesticide exposure is often driven more strongly by food- and water-related pathways than by inhalation. In addition, atmospheric pesticide levels are highly seasonal, meteorology-dependent, and less consistently monitored across countries.^48^ Thus, country-averaged air PI may be a relatively weak indicator for long-term cumulative cancer risk. In contrast, there was a significant positive correlation observed between PI in surface water and CR under advanced scenarios (Spearman r = 0.58; *p* < 0.05). The observed association can be attributed to the prominent contribution of water-related exposure pathways to the LADD under the advanced exposure scenario. In contrast to surface water, the association between PI in groundwater and CR under advanced scenarios was comparatively weak (Spearman r = 0.25, respectively; *p* > 0.1). This weaker relationship likely reflects the more limited exposure pathways associated with groundwater, which lacks the additional dermal and inhalation pathways characteristic of recreational usage compared with surface water.

These hotspots and pathway-specific findings also have direct implications for regulatory prioritization and public health management. In detail, interventions should be prioritized in hotspot regions according to the dominant exposure media and pathways. Where water-related pathways contributed strongly, countries should prioritize monitoring and control of pesticide residues in surface water, groundwater, and drinking-water systems. Where soil-related pathways contributed strongly, management should also focus on identifying and reducing exposure from legacy-contaminated soils. For countries with limited monitoring data, low estimated risk should not be assumed to indicate low true risk. A practical strategy is to establish sentinel monitoring in likely hotspot areas, prioritize high-persistence organochlorine pesticides, and use screening-level models before full monitoring coverage is available. Public health actions should pay particular attention to rural and agricultural communities and should incorporate legacy pesticide contamination into long-term cancer prevention strategies. Overall, the results support more integrated and risk-based management of environmental pesticides.

### Comparison with previous regional/national studies

To externally contextualize our estimates, we compared the spatial patterns identified in this study with previously published regional and national studies. However, due to the differences in scale, media, and methods, we only used prior studies for qualitative benchmarking and literature-based validation of hotspot location and dominant pathways rather than comparison of risk values.

At the global scale, our hotspot pattern is broadly consistent with previous large-scale studies. A recent global study used the Environmental Potential Risk Indicator for Pesticide model to calculate the predicted environmental concentration and then estimate the world geography of environmental pollution risk.^5^ The result found that some countries in Africa and Asia (e.g., South Africa, China, and India) are high-concern regions. This agrees well with our finding that many hotspot regions are located in Africa and Asia. Another study focused on global pesticide mixture risk in agricultural surface water^49^ successfully used a robust random forest model to identify the hotspots in Southern Asia and Africa. This finding highlights that water-related pathways become more important under the advanced scenario. In addition, a human health impact study from Europe also showed that pesticide-related health impacts vary strongly across substances, crops, and regions, although these studies did not produce country-level hotspot maps directly comparable to ours.^10^

For India and south Asia, a review of Punjab-specific evidence summarized pesticide residues in wheat flour, vegetables, human milk, and other food items, together with associated health concerns.^50^ A multi-compartment study from the Krishna river basin quantified twenty organochlorine pesticides in surface water, groundwater, and sediment, showing that historical pesticide inputs remain important and that sediment acts as a long-term sink.^51^ For China and east Asia, our results are also broadly aligned with prior regional studies, although the dominant medium varies by setting. In the Taihu lake region,^52^ organochlorine pesticides were widely detected in shallow groundwater, with DDTs and HCHs identified as the dominant contaminants, and the groundwater exposure may pose serious cancer risks to children.^53^ In contrast, a large survey of agricultural soils in the Pearl river delta found that DDTs and HCHs were again the major contributors,^53^ with higher soil burdens concentrated in multiple cities. For Mexico and Latin America, a study from northern Mexico has shown that organochlorine pesticides remain detectable in exposed populations and may contribute to carcinogenic risk.^54^ Taken together, these findings support the hotspot pattern in our result and that water- and soil-related pathways are important contributors to risk.

Overall, the benchmarking exercise suggests that our hotspot locations are generally consistent with published regional evidence, while the exact risk values are not expected to match one-to-one.

### Limitations of the study

Several limitations should be acknowledged in this study. First, the analysis only focused on carcinogenic pesticides with available toxicological parameters, excluding many new-generation pesticides and transformation products for which data remain scarce. As chemical substitution updates globally,^55^ future research should expand coverage to emerging compounds and their metabolites once their parameters are available. Second, although most pesticides evaluated in this study have been banned for decades, recent studies have continued to report detectable concentrations of these legacy pesticides in food items.^3^^,^^56^ Although the advanced scenario includes consumption of aquatic organisms from contaminated surface freshwater, it does not account for broader dietary exposure, such as crops, vegetables, fruits, and livestock products intake, which may underestimate the actual risks. The potential underestimation may be particularly important in agricultural communities where irrigation with contaminated water, pesticide residues in soils, and local food production can increase oral intake beyond the environmental pathways quantified here.^57^ Future research could extend the framework by integrating broader dietary exposure using food monitoring data, irrigation-related transfer, and food-consumption statistics. In addition, the mixture assessment assumed that cancer risks from different pesticides were independent, which is a common practice in cumulative risk assessment.^58^ However, this assumption may not fully capture possible interactions among chemicals, such as synergistic or antagonistic effects.^59^^,^^60^^,^^61^ Further progress in toxicological studies and mixture modeling would help reduce this uncertainty.^62^^,^^63^ For example, concentration addition, response addition, two-stage models, or quantitative structure-activity relationship approaches may offer improved prediction.^64^ However, these methods require extensive data on dose-response, interactions, or training. Given our global scale and data heterogeneity in this study, the independent-probability approach remains a pragmatic approximation. Recent studies^65^^,^^66^ suggest that artificial intelligence (AI) and machine-learning tools may support future pesticide risk assessment by improving toxicity prediction, prioritizing high-risk chemicals or mixtures, and assisting exposure modeling for data-sparse compounds. However, the usefulness of these tools still depends strongly on training data quality, interpretability, and regulatory acceptance. Future work could therefore combine mechanistic exposure modeling with AI-assisted screening to extend cumulative risk assessment to newer pesticides and transformation products. Also, this study calculated population-level environmental exposure, but did not explicitly account for different occupational exposures. For example, the environmental exposure doses among agricultural workers may be very high.^61^^,^^67^ This limitation is especially relevant in hotspot regions with intensive agricultural activity, where occupational and environmental exposures may co-occur to elevate cumulative risk.^68^ Future research could develop separate occupational exposure scenarios or worker-specific modules that can be linked to the present environmental background-risk framework. Moreover, country-level CR estimates are sensitive to the number and spatial coverage of pesticide concentration data; extremely high or low CR values may, in some cases, reflect limited monitoring data rather than true exposure conditions. For instance, data density was substantially higher in east Asia, Europe, and North America than in Africa and South America, which means that estimates in data-rich regions are likely to be more stable and may exert greater influence on the apparent global pattern. Conversely, underrepresented regions may contain unrecognized hotspots that are not fully captured by the current dataset. Therefore, low estimated risks in sparse-data regions should be interpreted as lower-confidence estimates rather than evidence of truly low risk.

Overall, future risk assessment frameworks would benefit from a more integrated approach, including the consideration of multiple exposure pathways, a broader range of new pesticides and their transformation products, and potential mixture effects. Such efforts would help improve cumulative cancer risk estimates and inform more effective long-term risk management strategies.

## Resource availability

### Lead contact

Further information and request for resources should be directed to and will be fulfilled by the lead contact, Zijian Li (lizijian3@mail.sysu.edu.cn).

### Materials availability

This study did not generate new unique materials.

### Data and code availability

• The pesticide pollution data were sourced from PestiGlobal: A Comprehensive Dataset on Pesticide Pollution and Standards (Figshare, https://doi.org/10.6084/m9.figshare.30271873).
• All calculations and analyses were performed using spreadsheet-based workflows in Microsoft Excel. The intermediate outputs required to reproduce the results are provided in the supplementary materials.
• Mapping and figure preparation were performed using ArcGIS (version 10.8) and R (version 4.5.0), based on the outputs generated from the spreadsheet workflows.
• This study does not report original code.
• Any additional information required to reanalyze the data reported in this study is available from the lead contact upon request.

## Acknowledgments

This study is financially supported by the 10.13039/501100001809National Natural Science Foundation of China (grant number, 32472598) and the Shenzhen Science and Technology Program (grant number, JCYJ20250604174437049).

## Author contributions

Y.H.: conceptualization, methodology, data curation, writing – original draft, and writing – review and editing. Z.L.: conceptualization, methodology, writing – review and editing, and funding acquisition.

## Declaration of interests

The authors declare no competing interests.

## Declaration of generative AI and AI-assisted technologies in the writing process

During the preparation of this work, the authors used ChatGPT in order to improve readability and language. After using this tool/service, the authors reviewed and edited the content as needed and take full responsibility for the content of the publication.

## STAR★Methods

### Key resources table

**Table:** 

REAGENT or RESOURCE	SOURCE	IDENTIFIER
**Deposited data**		

Dataset on Environmental Pesticide Residues (2010–2020)	Figshare	https://doi.org/10.6084/m9.figshare.30271873

**Software and algorithms**		

R 4.5.2	R Core Team	https://www.r-project.org/
ArcGIS 10.8	Environmental Systems Research Institute	https://www.arcgis.com/index.html
Microsoft Excel 2021	Microsoft	https://excel.cloud.microsoft/

**Other**		

Any additional information required to reanalyze the data reported in this paper	Available by request from lead contact	N/A

### Experimental model and study participant details

Omitted as our study does not involve biological models.

### Method details

#### Cancer risk assessment for a single pesticide via all possible exposure routes

We provided a quantitative method to assess the cancer risk of a single pesticide through all possible human exposure routes, including oral ingestion, inhalation, and dermal absorption. The approach integrates multimedia exposure assessment, internal dose estimation, and route-to-route extrapolation of CSFs to yield a unified, organ-specific cancer risk for each pesticide.

##### Step 1. Assessment of external chronic exposure doses

For each exposure route i, the lifetime average daily dose (*LADD*_*i*_, mg·kg^−1^·day^−1^) was calculated based on the established external exposure assessment model as follows:

(Equation 1) $${LADD}_{i}=\frac{C_{i}\times {IR}_{i}}{BW}$$

where *C*_*i*_ (mg·kg^−1^ or mg·L^−1^) denotes the pesticide concentration in the environmental media corresponding to the respective exposure route, and *IR*_*i*_ (kg·day^−1^ or L·day^−1^) denotes the respective intake rate (e.g., ingestion rate, inhalation rate, or dermal absorption rate). BW (kg) denotes the human body weight.

##### Step 2. Conversion of external to internal doses

External exposure doses were converted into internal pesticide concentrations in target organs using the physiologically based kinetic (PBK) modeling framework. The resulting internal dose (*C*_*Organ*_, mg·kg^−1^) represents the steady-state concentration of the pesticide in each organ due to chronic exposure through route *i* as follows:

(Equation 2) $$C_{Organ,i}=f_{PBK}\left(C_{i},k\in K\right)$$

where *f*_*PBK*_ denotes the PBK calculation function. *k*∈*K* denotes that the kinetic rate constant k belongs to the set K defined in the PBK model.

##### Step 3. Route-to-route extrapolation of cancer slope factors

CSFs are traditionally derived from oral studies, which link ingested doses to tumor incidence in specific organs. To enable integration across different exposure routes, inhalation and dermal exposures were converted to relative oral equivalents by comparing internal doses in the same target organs as follows^17^:

(Equation 3) $${CSF}_{i}={CSF}_{Oral}\times \frac{C_{Organ,i}}{C_{Organ,Oral}}$$

This route-to-route extrapolation assumes that tumor initiation and progression depend on the internal dose reaching the target organ, regardless of the original exposure route.

##### Step 4. Calculation of route-specific and aggregate cancer risks

For each exposure route i, the lifetime *CR*_*i*_ was estimated as follows:

(Equation 4) $${CR}_{i}={CSF}_{i}\times {LADD}_{i}$$

The overall cancer risk for the pesticide (*CR*_*Total*_) was then obtained by summing over all exposure routes as follows:

(Equation 5) $${CR}_{Total}=\sum_{i}^{3}{CR}_{i}$$

This aggregated cancer risk can reflect the aggregated carcinogenic potential arising from simultaneous exposures through multiple environmental pathways (i.e., air-, soil-, or water-based pathways).

#### Cancer risk assessment for pesticide mixtures via all possible exposure routes

Individuals are often simultaneously exposed to mixtures of multiple pesticides through various environmental media. To address this complexity, we extended the single-chemical cancer risk assessment method to account for mixtures of pesticides across all possible exposure routes (oral, inhalation, and dermal). This integrated approach quantifies route-specific internal doses for each chemical, determines organ-specific cancer responses, and aggregates risks under the assumption of statistical independence between tumor occurrences in different organs or mechanisms. Because different chemicals often target different organs, the total cancer risk for a mixture was estimated using the independent-probability approach. Assuming tumor occurrence in different organs or tissues represents independent events, the cumulative probability of developing at least one tumor from the entire pesticide mixture is given by^69^:

(Equation 6) $${CR}_{Total,Mixture}=1-\prod_{j}^{J}\left(1-{CR}_{Total,j}\right)=1-\prod_{j}^{J}\left(1-\sum_{i}^{3}{CR}_{i,j}\right)$$

where *CR*_*Total*, *Mixture*_ denotes the overall cancer risk caused by the pesticide mixture via all possible exposure routes. *CR*_*Total*,*j*_ denotes the overall cancer risk caused by pesticide j via all possible exposure routes. *CR*_*i*,*j*_ denotes the cancer risk caused by pesticide j via exposure route i.

#### Global pesticide pollution data

To establish a unified global resource on pesticide contamination, we systematically gathered observational data on pesticide residues across key environmental media (air, freshwater, and residential soil). A structured literature search was performed for the period 2010–2020 using international databases such as Web of Science, Scopus, CNKI, and PubMed. Keywords included combinations of pesticide, residue, monitoring, environmental concentration, air, soil, and water. For each record, pesticide name, sampling location, sampling year, environmental medium, measured concentration, detection frequency, and analytical method were extracted. All concentration values were converted into uniform SI units. To ensure cross-comparability, pesticide names were standardized according to the CAS registry numbers. When a database provided only geographical coordinates (latitude and longitude) of sampling sites without explicit administrative information, the corresponding country and subnational administrative division (e.g., province or state) were identified using Google Maps.

The curated dataset covers observations from over one hundred countries and regions.^70^ Data density was highest in East Asia, Europe, and North America, reflecting the geographic distribution of existing monitoring programs. Temporal coverage spans 2010–2020, providing a contemporary overview of global pesticide pollution dynamics during the past decades.

#### Global cancer risk assessment

##### Integration of environmental media and exposure pathways

To quantify population cancer risks, pesticide residue data were integrated across four environmental media (air, soil, surface freshwater, and groundwater), each linked to specific exposure pathways. All exposure routes were categorized as inhalation, oral ingestion, or dermal contact, depending on the medium and human activity pattern.

(a) Medium-based exposure pathways

Air: inhalation of airborne pesticides and dermal contact with air particulates.

Soil: oral ingestion of soil particles, inhalation of soil dust, and dermal contact with soil.

Surface freshwater (rivers, lakes, reservoirs): ingestion of drinking water, recreational exposure (incidental water ingestion, inhalation, and dermal absorption during swimming, boating, or fishing), ingestion of aquatic organisms (consumption of fish or shellfish accumulating pesticides from surface water), and showering exposure (dermal absorption and inhalation of pesticide volatilized from municipally supplied water).

Groundwater: ingestion of drinking water and showering exposure (dermal and inhalation exposure during domestic use of contaminated groundwater).

(b) Definition of exposure scenarios
  To accommodate variability in data availability and population activities, two exposure scenarios were defined.
  (b1) Basic scenario (common assessment)
    This scenario represents typical environmental exposure conditions. It includes:
    Air: inhalation and dermal contact.
    Soil: ingestion of soil particles, inhalation of soil dust, and dermal contact.
    Freshwater (surface and groundwater): drinking water ingestion only.
    This approach ensures coverage of the most consistent and routinely monitored exposure pathways, suitable for regional or national risk screening where only partial media data are available.
  (b2) Advanced scenario (worst-case comprehensive assessment)

The advanced scenario incorporates all possible human and environmental interaction pathways, reflecting high-exposure or worst-case conditions. It includes:

All air and soil pathways listed above; All surface freshwater and groundwater pathways, including recreational contact, fish consumption, and showering exposure.

This scenario provides an upper-bound estimate of cancer risk for populations living in regions where pesticides are extensively used or where multimedia contamination is likely to co-occur. The detailed equations and parameters were listed in the Supplementary Word File (Tables S8 and S9).

#### Regionalization of population exposure

Because environmental monitoring data are spatially heterogeneous, defining a realistic “population region” for exposure assessment is essential. In this study, cancer risk assessments were primarily performed at the national level. However, for countries with a total land area greater than 2,300,000 km^2^, the assessments were performed at the provincial or state level, as substantial intra-national heterogeneity was expected. This spatial scale balances environmental representativeness and population data availability. The following aggregation logic was applied to harmonize data across cities within the same administrative region.

##### City-level averaging

When two or more cities within the same province reported pesticide concentrations for the same medium and pesticide, the simple arithmetic mean was used if sample numbers were not reported. When sample numbers were available, a weighted mean was calculated using the number of samples as weights.

##### Data sharing across cities

If one city within the province had available pesticide data while neighboring cities lacked data for the same pesticide and medium, those cities were assumed to share the same regional value. This assumption is justified by the similar emission patterns, climate, and agricultural practices expected within a single administrative province or state. When reported data from a study covering multiple provinces or states jointly (i.e., cross-provincial data), the same concentration values were shared across all involved administrative regions to maintain comparability in exposure estimation.

##### Multi-city aggregation

For provinces with multiple cities reporting data across different media, the province-level concentration for each pesticide and medium was determined by averaging across all contributing cities (weighted when possible). This process ensures internal consistency and mitigates spatial gaps.

##### Cross-media harmonization

After provincial aggregation, air, soil, and water datasets were combined according to the exposure pathways defined in the basic and advanced scenarios. This yielded unified provincial-level pesticide concentration matrices, which were then used as inputs for exposure and cancer risk modeling.

### Quantification and statistical analysis

Spearman’s rank correlation was used to evaluate associations between pesticide PI^7^^,^^47^ and country-level averaged CR values, with statistical significance set at *p* < 0.05. Correlation coefficients, exact two-sided *p* values, and sample sizes are reported in Table S7; the corresponding scatterplots are shown in Figure S1.

To evaluate the robustness of the estimated CR values, we conducted three sensitivity analyses addressing (1) parameter uncertainty, (2) regional data gaps, and (3) data aggregation assumptions. In all three analyses, the advanced-scenario TCR was recalculated because it represented the most comprehensive exposure scenario.

First, to evaluate the robustness of the estimated cancer risks, we assigned lower-bound and upper-bound values to major uncertain inputs, including selected exposure-related parameters to key uncertain inputs, including exposure-related parameters affecting LADD and PBK parameters. A one-at-a-time sensitivity analysis was then performed to identify the dominant contributors to model uncertainty. Second, to assess the influence of regional data gaps, we generated two restricted datasets: (i) a moderate-coverage subset including only countries/regions supported by at least two independent monitoring studies or environmental compartments, and (ii) a high-coverage subset including only countries/regions supported by at least three independent studies or environmental compartments. Advanced TCRs were recalculated for these datasets and compared with the baseline analysis using hotspot overlap and median risk values. Third, to assess the impact of spatial smoothing due to multi-province/state data sharing, we performed a sensitivity analysis excluding all pooled multi-province/state observations. In this scenario, only province/state-specific data (including city/site-level observations) were retained, and province/state-level concentrations were recalculated following the same aggregation procedure as the baseline. Similarly, the resulting advanced-scenario TCR estimates were compared with baseline results using median TCR and hotspot overlap. Sensitivity settings and outputs for key parameters, data gaps, and data aggregation are provided in Tables S3, S4, and S5.

## Supplemental information

Document S1. Figure S1 and Tables S8 and S9

Table S1. Calculation of LADD and CR

Table S2. Summary of CR values

Table S3. Sensitivity analysis of key parameters

Table S4. Sensitivity analysis of data gaps

Table S5. Sensitivity analysis of data aggregation

Table S6. CSF conversion values for risk assessment

Table S7. Correlation between PI and CR
