# Supplementary material for: Mapping global cancer risk hotspots from environmental pesticides
Source: iScience. 2026 Jul 7;29(7):116599. doi: 10.1016/j.isci.2026.116599 (PMC13378364; doi:10.1016/j.isci.2026.116599)
Supplement: Document S1. Figure S1 and Tables S8 and S9 [file mmc1.pdf]

iScience, Volume *29*

Supplemental information

Mapping global cancer risk hotspots from environmental pesticides

Yabi Huang and Zijian Li

Table S8. All exposure equations describing human intake from soil, air, and water[1–3].

**Exposure pathways**	**Definitions**
	**Air**
Inhalation of airborne pesticides	$LADD_{Air,Inha} = C_{Air} \times \left(\frac{\mathrm{AIR}}{\mathrm{BW}}\right)$
Dermal contact with gas phase	$LADD_{Air,Derm} = C_{Air} \times \mathrm{k}_p \times t_{Air} \times \left(\frac{\mathrm{SA}}{\mathrm{BW}}\right) \times CF_1$
	**Soil**
Oral ingestion of soil particles	$LADD_{Soil,Oral} = C_{Soil} \times \left(\frac{\mathrm{ED_{Soil}EDF_{Soil}EFR_{Soil}}}{365\mathrm{LT}}\right) \times \left(\frac{\mathrm{IR}_{Soil}}{\mathrm{BW}}\right)$
Inhalation of soil dust	$LADD_{Soil,Inhal} = C_{Soil} \times (\frac{1}{\mathrm{PRF}}) \times \left(\frac{\mathrm{ED_{Soil}EDF_{Soil}EFR_{Soil}}}{365\mathrm{LT}}\right) \times \left(\frac{AIR}{\mathrm{BW}}\right) \times CF_2$
Dermal contact with soil	$LADD_{Soil,Derm} = C_{Soil} \times \mathrm{DA_{Event,soil}} \times \left(\frac{\mathrm{ED_{Soil}EF_{Soil}EFR_{Soil}}}{365\mathrm{LT}}\right)\left(\frac{\mathrm{SA}}{\mathrm{BW}}\right) \times CF_1$
	**Surface freshwater (rivers, lakes, reservoirs)**
Ingestion of drinking water	$LADD_{Overall,DWR} = C_{SW} \times \mathrm{TF} \times \left(\frac{\mathrm{IR_{DW}}}{\mathrm{BW}}\right)$
Recreational exposure	Incidental water ingestion, inhalation, and dermal absorption during swimming, boating, or fishing<br>$LADD_{Overall,Recreation} = LADD_{Oral,Swim} + LADD_{Inha,Swim} + LADD_{Derm,Swim} + LADD_{Inha,Boating} + LADDF_{Inha,Fishing}$<br>$LADD_{Oral,Swim} = C_{SW} \times \left(\frac{\mathrm{ED_{SW}EDF_{SW}EFR_{SW}}}{365\mathrm{LT}}\right) \times \left(\frac{24\mathrm{IR_{SW}}}{\mathrm{BW}}\right)$

	$LADD_{Inha,Swim} = C_{SW} \times \mathrm{K_{AW}} \times \left(\frac{\mathrm{ED_{SW}EDF_{SW}EFR_{SW}}}{\mathrm{365LT}}\right) \times \left(\frac{\mathrm{AIR_{SW}}}{\mathrm{BW}}\right)$<br>$LADD_{Derm,Swim} = C_{SW} \times \mathrm{DA_{Event,SW}} \times \left(\frac{\mathrm{ED_{SW}EF_{SW}EFR_{SW}}}{\mathrm{365LT}}\right) \times \left(\frac{\mathrm{SA}}{\mathrm{BW}}\right)$<br>$LADD_{Inha,Boating} = C_{SW} \times \mathrm{K_{AW}} \times \left(\frac{\mathrm{ED_{SP}EDF_{SP}EFR_{SP}}}{\mathrm{365LT}}\right) \times \left(\frac{\mathrm{AIR_{SP}}}{\mathrm{BW}}\right)$<br>$LADD_{Inha,Fishing} = C_{SW} \times \mathrm{K_{AW}} \times \left(\frac{\mathrm{ED_{FI}EDF_{FI}EFR_{FI}}}{\mathrm{365LT}}\right) \times \left(\frac{\mathrm{AIR_{FI}}}{\mathrm{BW}}\right)$
Ingestion of aquatic organisms	consumption of fish or shellfish accumulating pesticides from surface water<br>$LADD_{Oral,Aquatic} = C_{SW} \times \mathrm{BCF_{Fish}} \times \left(\frac{\mathrm{IR_{FI}}}{\mathrm{BW}}\right) + C \times \mathrm{BCF_{Shellfish}} \times \left(\frac{\mathrm{IR_{FI}}}{\mathrm{BW}}\right)$
Showering exposure	Dermal absorption and inhalation of pesticide volatilized from municipally supplied water<br>$LADD_{Overall,Shower} = LADD_{Inha,SH} + LADD_{Derm,SH})$<br>$LADD_{Inha,SH} = C_{SW} \times \mathrm{K_{AW}} \times \left(\frac{\mathrm{ED_{SH}EDF_{SH}EFR_{SH}}}{\mathrm{365LT}}\right) \times \left(\frac{\mathrm{AIR_{SH}}}{\mathrm{BW}}\right)$<br>$\mathrm{LADD_{Derm,SH}} = C_{SW} \times \mathrm{DA_{Event,SH}} \times \left(\frac{\mathrm{ED_{SH}EF_{SH}EFR_{SH}}}{\mathrm{365LT}}\right) \times \left(\frac{\mathrm{SA}}{\mathrm{BW}}\right)$
**Groundwater**	
Ingestion of drinking water	$LADD_{Oral,DW} = C_{GW} \times \mathrm{TF} \times \left(\frac{\mathrm{IR_{DW}}}{\mathrm{BW}}\right)$
Showering exposure	Dermal and Inhalation exposure during domestic use of contaminated groundwater<br>$LADD_{Overall,Shower} = LADD_{Inha,SH} + LADD_{Derm,SH}$

	$LADDF_{Inha,SH} = C_{GW} \times \mathrm{TF} \times \mathrm{K_{AW}} \times \left(\frac{\mathrm{ED_{SH}EDF_{SH}EFR_{SH}}}{365\mathrm{LT}}\right) \times \left(\frac{\mathrm{AIR_{SH}}}{\mathrm{BW}}\right)$ $LADDF_{Derm,SH} = C_{GW} \times \mathrm{TF} \times \mathrm{DA_{Event,SH}} \times \left(\frac{\mathrm{ED_{SH}EF_{SH}EFR_{SH}}}{365\mathrm{LT}}\right) \times \left(\frac{\mathrm{SA}}{\mathrm{BW}}\right)$

Table S9. Summary of exposure parameters.

Variables	Unit	Definitions	Values	Reference
AIR	L d^{-1}	inhalation rate	12000	4
1/BW	kg^{-1}	body weight	0.0161	2
K_P	cm hr^{-1}	dermal permeability coefficient	It differs from the pesticides	2
SA	cm^2	skin area available for contact	16776.32	2
ABS	unitless	absorption coefficient for skin, indicating the skin absorption coefficient	0.1	5
ED_{Soil}	yr	exposure duration	50	5
EDF_{Soil}	unitless	effective day factor	0.25	5
EER_{Soil}	d yr^{-1}	exposure frequency	350	5
IR_{Soil}	kg d^{-1}	intake rate for soil	100	5
LT	yr	average exposure lifetime	70.00	2
PRF	m^3 kg^{-1}	powder generation factor, indicating the soil dust generation coefficient	0.00001	5
$DA_{Event,soil}$	mg cm^{-2} $event^{-1}$	absorption dose per event	It differs from the pesticides	2
TF	unitless	treating factor	1	2
ED_{SW}	yr	exposure duration for swimming	50.00	2
ED_{SP}	yr	exposure duration for sports like boating	50.00	2
ED_{FI}	yr	exposure duration for fishing	50.00	2
ED_{SH}	yr	exposure duration for showering	70.00	2
EDF_{SW}	unitless	effective day factor for swimming	0.0625	2
EDF_{SP}	unitless	effective day factor for sports like boating	0.0660	2
EDF_{FI}	unitless	effective day factor for fishing	0.25	2
EDF_{SH}	unitless	effective day factor for showering	365.00	2
EER_{SW}	d yr^{-1}	exposure frequency for swimming	120.00	2
EER_{SP}	d yr^{-1}	exposure frequency for sports like boating	17.00	2

EER_{FI}	d yr^{-1}	exposure frequency for fishing	17.00	2
EER_{SH}	d yr^{-1}	exposure frequency for showering	365	2
IR_{DW}	L d-	intake rate for drinking water	2.00	2
IR_{SW}	L hr^{-1}	intake rate for swimming	0.02	2
IR_{FI}	kg d^{-1}	intake rate for fish	0.02	2
$IR_{Shellfish}$	kg d^{-1}	intake rate for shellfish	0.0035	6
AIR_{SW}	L d^{-1}	inhalation rate for showing	76800.00	2
AIR_{SP}	L d^{-1}	inhalation rate for sports like boating	76800.00	2
AIR_{FI}	L d^{-1}	inhalation rate for fishing	15700.00	4
AIR_{SH}	L d^{-1}	inhalation rate for showering	15700.00	4
$DA_{Event,SH}$	mg cm^{-2} event^{-1}	absorption dose per event for showing	It differs from the pesticides	2
$DA_{Event,SW}$	mg cm^{-2} event^{-1}	absorption dose per event for swimming	It differs from the pesticides	2
K_{AW}	unitless	air–water partition coefficient	It differs from the pesticides	2,7
BCF	unitless	bioconcentration factor of chemicals in fish	It differs from the pesticides	2,8,9

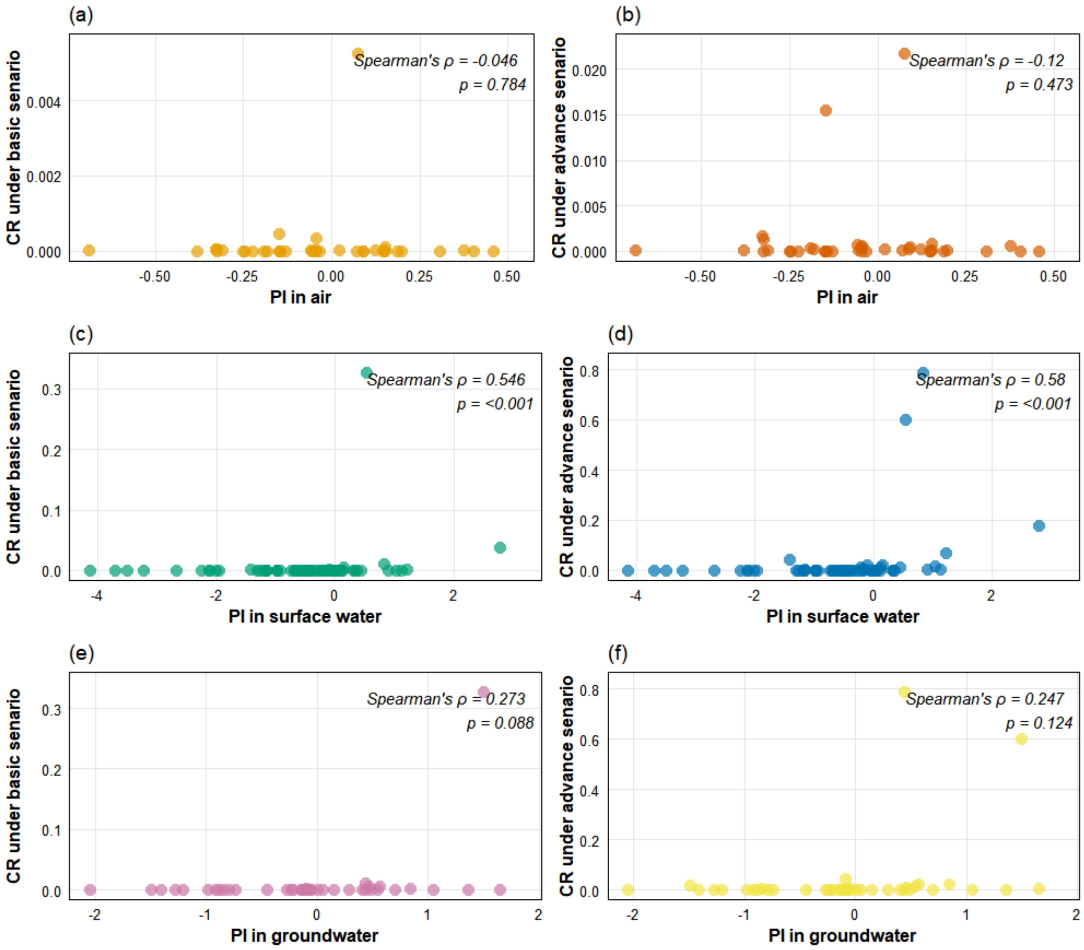

Figure S1. Scatter plots showing the relationships between pesticide Pollution Index (PI) and country-level cancer risk (CR) across air, surface water, and groundwater under the basic (a,c, and e) and advanced exposure scenarios (b,d and f).

Reference

1. Xiong, J. & Li, Z. Predicting PFAS fate in fish: Assessing the roles of dietary, respiratory, and dermal uptake in bioaccumulation modeling. *Environ Res* 252, 119036 (2024).

2. Xiong, J., Li, Z. & Fantke, P. Modeling Human Exposure to Chemicals Via Different Freshwater Pathways. *Expo Health* https://doi.org/10.1007/s12403-025-00702-7 (2025) doi:10.1007/s12403-025-00702-7.

3. Huang, Y., Li, Z. & Fantke, P. Defining pesticide water quality standards for multi-use water resources to protect human health. *Sci Total Environ* 999, 180344 (2025).

4. USEPA. Exposure Factors Handbook. https://www.nrc.gov/docs/ML1015/ML101590178.pdf (1997).

5. USEPA. Superfund Soil Screening Guidance. https://www.epa.gov/superfund/superfund-soil-screening-guidance (2002).

6. FAO. The State of World Fisheries and Aquaculture 2018. *2018* https://openknowledge.fao.org/server/api/core/bitstreams/6fb91ab9-6cb2-4d43-8a34-a680f65e82bd/content (2018).

7. USEPA. Regional Screening Levels (RSLs) - Chemical Specific Parameters. https://www.epa.gov/risk/regional-screening-levels-rsls-generic-tables (2018).

8. Arnot, J. A. & Gobas, F. A. A review of bioconcentration factor (BCF) and bioaccumulation factor (BAF) assessments for organic chemicals in aquatic organisms. *Environ Rev* 14, 257–297 (2006).

9. Przybyla J, Klotzbach J M, Salinas K, Citra, M. & Crisman, J. S. *Toxicological Profile for 1,1,2,2-Tetrachloroethane*. (2021).
